# Cannabis vapor self-administration elicits sex- and dose-specific alterations in stress reactivity in rats

**DOI:** 10.1016/j.ynstr.2020.100260

**Published:** 2020-10-18

**Authors:** Nicholas C. Glodosky, Carrie Cuttler, Timothy G. Freels, Hayden R. Wright, Manuel J. Rojas, Samantha L. Baglot, Matthew N. Hill, Ryan J. McLaughlin

**Affiliations:** a Department of Psychology, Washington State University, Pullman, WA, USA; b Department of Integrative Physiology and Neuroscience, Washington State University, Pullman, WA, USA; c Animal Health Department, Universidad Nacional de Colombia, Bogotá, Colombia; d Departments of Cell Biology and Anatomy and Psychiatry, Hotchkiss Brain Institute, University of Calgary, Calgary, AB, Canada

**Keywords:** Cannabis, Vapor, THC, Stress, Corticosterone, Self-administration, Animal model

## Abstract

**Rationale:**

Cannabis users frequently report stress relief as their primary reason for use. Recent studies indicate that human cannabis users exhibit blunted stress reactivity; however, it is unknown whether this is a cause or a consequence of chronic cannabis use.

**Objectives:**

To determine whether chronic cannabis vapor self-administration elicits sex- and/or dose-dependent alterations in stress reactivity and basal corticosterone (CORT) concentrations, or whether pre-vapor exposure stress reactivity predicts rates of cannabis vapor self-administration.

**Methods:**

Male and female rats were subjected to 30 min acute restraint stress to assess stress reactivity prior to vapor self-administration. Rats were then trained to self-administer cannabis extract vapor containing 69.9% Δ^9^-tetrahydrocannabinol (THC) at one of four extract concentrations (0, 75, 150, or 300 mg/ml) daily for 30 days. Half of the rats were then subjected to a second restraint stress challenge 24 h after the final self-administration session, while the other half served as no-stress controls. Plasma CORT concentrations were measured prior to stress and immediately post-stress offset.

**Results:**

Female rats earned significantly more vapor deliveries than male rats. Pre-vapor stress reactivity was not a predictor of self-administration rates in either sex. Basal CORT concentrations were increased following vapor self-administration relative to pre-vapor assessment, irrespective of treatment condition. Importantly, cannabis self-administration dose-dependently reduced stress reactivity in female, but not male, rats.

**Conclusions:**

These data indicate that chronic cannabis use can significantly dampen stress reactivity in female rats and further support the use of the cannabis vapor self-administration model in rats of both sexes.

## 1 Introduction

The shifting social and political landscape surrounding cannabis has been associated with an increase in daily cannabis use among adults (Mauro et al., 2018). Cannabis users frequently cite stress relief as their primary reason for using cannabis (Copeland et al., 2001; Green et al., 2003; Hyman and Sinha 2009) and they report using cannabis for coping with negative affect and life problems more than any other drug (Green et al., 2003). Consistent with this, acute cannabis use has been shown to reduce perceived stress (Cuttler et al., 2018). Moreover, oral administration of Δ^9^ tetrahydrocannabinol (THC), the primary psychoactive constituent of cannabis, produces dose-dependent reductions in ratings of distress following exposure to an acute stressor compared to placebo administration (Childs et al., 2017). These dose-dependent effects of acute cannabis use are generally well accepted. Nevertheless, acute THC administration has also been shown to increase concentrations of cortisol among cannabis users compared to baseline (D'Souza et al., 2004; Ranganathan et al., 2009) or to a placebo control condition (Klumpers et al., 2012). This is similar to alterations in cortisol release that have been observed among nicotine and alcohol users, who display increased cortisol concentrations after acute consumption, as well as diminished cortisol reactivity to stress after chronic use (Lovallo, 2006).

The extent to which chronic cannabis use alters the stress response, however, remains less well known. This is surprising given that the endocannabinoid (ECB) system, which is the primary target for THC, is fundamentally involved in regulation of the neuroendocrine stress response (see Morena et al., 2016 for review). A handful of studies have indicated that chronic cannabis users exhibit higher basal (Carol et al., 2017; King et al., 2011; Monteleone et al., 2014; Somaini et al., 2012) and awakening (Huizink et al., 2006) cortisol concentrations compared to non-users. Sober chronic cannabis users also display blunted amygdala activation and reduced emotional reactivity to emotionally-laden stimuli (Cornelius et al., 2010), as well as dampened adrenocorticotropic hormone (ACTH) and cortisol release in response to unpleasant images (Somaini et al., 2012). Accordingly, we have recently shown that sober chronic cannabis users exhibit a blunted stress response relative to non-users (Cuttler et al., 2017). In this study, chronic cannabis users and non-users were randomly assigned to receive a multidimensional stressor or no stress control condition and were asked to rate their subjective level of stress and provide saliva samples to measure cortisol before and after the stress manipulation. While the non-cannabis users in the stress condition demonstrated the expected increase in cortisol relative to non-users in the control condition, the change in cortisol for the cannabis users in the stress condition was not significantly different than the change in cortisol for cannabis users in the no-stress condition. Similarly, cannabis users showed a diminished increase in subjective stress ratings compared to non-users (Cuttler et al., 2017). Thus, chronic cannabis use may alter components of the neuroendocrine stress axis and interfere with the ability of the ECB system to modulate the stress response, even under drug-free conditions.

A major limitation with this and other studies exploring effects of chronic cannabis use in humans is that chronic cannabis use cannot be ethically experimentally manipulated. As such, causal conclusions cannot be established, and it is therefore unclear whether the effects observed in human studies were caused by regular cannabis use, or otherwise due to pre-existing differences in the stress response that increase propensity for habitual cannabis use. In line with this alternative explanation, a previous study has indicated that sons of fathers with a substance use disorder showed decreased cortisol reactivity to stress, which was later associated with regular monthly use of cannabis during adolescence (Moss et al., 1999).

Animal models of cannabis use provide a means to systematically manipulate cannabis use while controlling for extraneous factors that often complicate data interpretation. Despite the advantages of using animal models to experimentally manipulate cannabis use, there have historically been limitations with preclinical cannabis studies that decrease their translational relevance. For instance, research exploring effects of cannabis in animal models often do not use cannabis, but rather use synthetic CB1 receptor agonists or isolated constituents of cannabis, which may produce different effects (McLaughlin 2018). The use of intraperitoneal or intravenous delivery of cannabinoids is also popular in rodent models, even though these are uncommon routes of administration in humans due to the likelihood of adverse psychological events (Carbuto et al., 2012; D'Souza et al., 2004). Further, there is considerable variability in the pharmacokinetics of cannabinoids depending on the route of administration (Grotenhermen 2003; Hložek et al., 2017; Huestis 2007). With this in mind, our research group has recently developed and validated a novel vapor exposure model that employs response-contingent administration of vaporized cannabis extracts of varying concentrations (Freels et al., 2020). We have shown that vaporized cannabis extracts rich in THC produce biologically and behaviorally relevant plasma cannabinoid concentrations, have robust motivational properties, and support conditioned responding for cannabis-paired cues following a period of abstinence (Freels et al., 2020). This model is particularly advantageous from a translational perspective because inhalation is the most common route of administration among human cannabis users (Sexton et al., 2016).

We used this novel vapor self-administration approach in the current study to determine the direction of the relationship between cannabis use and blunted stress reactivity. Specifically, our primary objective was to examine whether chronic cannabis vapor self-administration alters basal and/or stress-induced corticosterone (CORT) concentrations in male and female rats, or alternately whether stress reactivity prior to cannabis exposure is a significant predictor of rates of cannabis vapor self-administration. Additionally, since our recent study only examined vapor self-administration in male rats and did not compare rates of self-administration across different extract concentrations (Freels et al., 2020), we also compared responding in age-matched male and female rats using a range of cannabis extract concentrations. In line with human data, we predicted that rats trained to self-administer high concentrations of cannabis vapor will display elevated basal CORT and blunted stress reactivity during an acute stress challenge under drug-free conditions. Moreover, given that both human and animal literature indicate sex-dependent effects of cannabinoid exposure (see Cooper and Craft 2018 for a review), and pronounced sex differences in the stress response (Gunn et al., 2016), we further hypothesized that rates of cannabis vapor self-administration will differ between sexes and that cannabis vapor self-administration will elicit dose- and sex-dependent changes in basal and stress-induced CORT relative to vehicle self-administration.

## 2 Materials and methods

### 2.1 Subjects

**Fig. 1:**
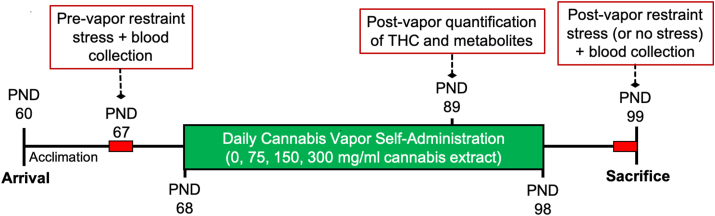
**Illustration of experimental timeline.** Acute stress challenges are indicated with red blocks, while vapor self-administration is indicated by the green block. (For interpretation of the references to colour in this figure legend, the reader is referred to the Web version of this article.)

Adult Sprague Dawley rats (*N* = 104 [*n* = 13/sex/condition]) were received from Simonsen Laboratories (Gilroy, CA) at postnatal day 60. All rats were housed in same-sex pairs in standard polycarbonate cages and given *ad libidum* access to food and water for the duration of the study. Rats were kept on a 12-h reverse light-dark cycle (lights off at 7h00) such that all procedures were conducted during the rats’ active phase. Rats were given one week of acclimation and handling prior to initiation of the study. Experiments were run in 3 independent cohorts, but importantly, each cohort contained equal numbers from each sex and treatment group and all testing was conducted at the same time of day to mitigate potential cohort or time of day effects, respectively. All procedures followed the National Institutes of Health Guide for the Care and Use of Laboratory Animals and were approved by the Washington State University Institutional Animal Care and Use Committee. An experimental timeline detailing the procedure is provided in Fig. 1.

### 2.2 Apparatus and materials

#### 2.2.1 Drugs

A raw cannabis extract containing 69.81% THC was obtained from the National Institute on Drug Abuse (NIDA) Drug Supply Program. According to the certificate of analysis, this extract also contained 0.89% tetrahydrocannabivarin (THCV), 0.83% cannabichromene (CBC), 2.69% cannabigerol (CBG), 1.51% cannabinol (CBN), and 0.73% Δ^8^ tetrahydrocannabinol (Δ^8^ THC). Cannabidiol (CBD) concentration was below the threshold of detection. The total terpene concentration present in the extract was 1.35% (information regarding specific terpenes was not available). The extract was heated to 60 °C for 15–20 min under constant stirring and suspended in an 80% propylene glycol/20% vegetable glycerol vehicle (PG/VG) at cannabis extract concentrations of 0 mg/ml (VEH), 75 mg/ml, 150 mg/ml, and 300 mg/ml (mg extract/ml vehicle). This dose range was chosen based on the THC concentrations used by our group (Freels et al., 2020) and others (Nguyen et al., 2016). Final concentrations of THC were 0 mg/ml in the vehicle, 52.5 mg/ml in the 75 mg/ml preparation, 105 mg/ml in the 150 mg/ml preparation, and 210 mg/ml in the 300 mg/ml preparation (mg THC/ml suspension).

#### 2.2.2 Vapor self-administration

A 16-chamber vapor delivery system (14.5″ L x 10.5″ W x 9.5” H; La Jolla Alcohol Research Inc. [LJARI]) programmed using MED-Associates IV or LJARI software was used to deliver response-contingent puffs of vapor on a fixed ratio-1 (FR-1) schedule of reinforcement during daily 1 h sessions for the duration of the 30 days of self-administration, as described in Freels et al. (2020). Vapor self-administration sessions were conducted at the same time each day, between 8h00 and 11h00. Briefly, a commercial e-cigarette tank (SMOK Tank Baby Beast TFV8 with 0.25Ω M2 atomizer, Shenzhen, China) was filled with cannabis extract at the concentrations specified above. Two nosepoke operanda, each with an associated cue light, were also located at the back of the chamber. Operanda were assigned as active or inactive, and when a response was made on the active operanda, a 3 s puff of vapor was delivered into the chamber. Delivery of vapor was paired with illumination of the associated cue light, which remained illuminated for 60 s while the vapor remained in the chamber. Responses made on the inactive operanda or on the active port during the 60 s timeout were recorded but had no programmed consequences. Vapor was evacuated via a vacuum pump at the back of the chamber connected to an in-line activated charcoal filter (Millipore-Sigma, St. Louis, MI).

#### 2.2.3 Quantification of THC and metabolites

Deuterated Δ^9^-tetrahydrocannabinol (THC), 11-hydroxy-THC (11-OH-THC) and 11-nor-9-carboxy-THC (THC-COOH; all purchased from Cerilliant [Round Rock, TX, USA]) were dissolved in acetonitrile at a concentration of 1.0 mg/mL. An internal standard (IS; d3 analytes) solution contains each compound at 10 ng/ml and was prepared in 50% methanol/water. Glass tubes containing 2 mL of acetonitrile and 100 μL of IS were prepared to receive plasma and brain samples. Each plasma sample was thawed at room temperature and 50 μL was directly pipetted into the prepared tubes. All samples were then sonicated in an ice bath for 30 min before being stored overnight at −20 °C to precipitate proteins. The next day samples were centrifuged at 1800 rpm at 4 °C for 3–4 min to remove particulates and the supernatant from each sample was transferred to a new glass tube. Tubes were then placed under nitrogen gas to evaporate. Following evaporation, the tube sidewalls were washed with 250 μL acetonitrile in order to recollect any adhering lipids and then again placed under nitrogen gas to evaporate. Following complete evaporation, the samples were re-suspended in 100 μL of 1:1 methanol and deionized water. Resuspended samples went through two rounds of centrifugation (15,000 rpm at 4 °C for 20 min) to remove particulates and the supernatant transferred to a glass vial with a glass insert. Samples were then stored at −80 °C until analysis by LC-MS/Multiple Reaction Monitoring (MRM) on an Eksigent Micro LC200 coupled to an AB Sciex QTRAP 5500 mass spectrometry (AB Sciex, Ontario, Canada) at the Southern Alberta Mass Spectrometry (SAMS) facility located at the University of Calgary. The data were acquired in positive electrospray ionization (ESI) and multiple reaction monitoring (MRM) mode. Analyte concentration (in pmol/μL) were normalized to sample volume and converted to ng/mL for presentation.

#### 2.2.4 Acute restraint stress

Acute restraint stress was conducted for 30 min in all rats prior to vapor exposure using Broome Rodent Restrainers (2.28–7.68″ L x 2.5” H, Harvard Apparatus, Holliston, MA) (pre-vapor stress challenge). Twenty-four hours after the final vapor self-administration session (between 8h00 and 11h00), roughly half of each group of rats was exposed to a second 30 min acute restraint stress challenge (post-vapor stress challenge). Specifically, in the 0 and 75 mg/ml conditions 14 rats (7 per sex) were exposed to the stress challenge and 12 (6 per sex) remained in their home cage and served as no-stress controls. In the 150 and 300 mg/ml conditions 12 rats (6 per sex) were exposed to the stressor and 14 (7 per sex) served as no-stress controls. Blood was collected prior to and immediately after each stress exposure via the tail vein in sterile 10 ml tubes containing 0.1 ml ethylenediaminetetraacetic acid, centrifuged at 4 °C at 4000 g for 15 min, and stored at −20 °C. All sample collection was performed at the same time of day for all experimental groups.

#### 2.2.5 Corticosterone measurements

Commercially available enzyme-linked immunosorbent assay (ELISA) plasma CORT test kits (Arbor Assays, Ann Arbor, MI) were used to quantify plasma concentrations of CORT according to manufacturer instructions.

### 2.3 Statistical analyses

Data were screened for outliers (i.e., values exceeding ± 3.29 standard deviations from the mean). Outliers (<0.5% of data) were trimmed to one unit higher or lower than the nearest nonoutlying raw score value (Tabachnick and Fidell 2013). Alpha was set at 0.05 (two-tailed) for all analyses.

#### 2.3.1 Vapor self-administration

Four separate 2 × 4 × 30 mixed factorial analyses of variance (ANOVAs) were conducted with sex, treatment, and time as independent variables and active-port responses, vapor deliveries, inactive-port responses, and the discrimination index as dependent variables. Discrimination index was calculated using the formula: $DI=\frac{active\;nosepokes\;-\;inactive\;nosepokes}{active\;nosepokes\;+\;inactive\;nosepokes}$, such that 0 indicates no discrimination between the active and inactive port, 1 indicates perfect active-port responding, and −1 indicates perfect inactive-port responding (Freels et al., 2020). The assumption of sphericity was violated in these analyses, so Greenhouse-Geisser corrected statistics are reported. Interactions with sex were probed by conducting follow-up 4 × 30 ANOVAs examining effects of treatment and time on responding in males and females separately. Bonferroni post hoc analyses were conducted to probe significant main effects of treatment.

#### 2.3.2 Plasma THC and metabolite concentrations

Three 2 × 3 between group ANOVAs with sex and treatment as independent variables and levels of THC and metabolites (THC-COOH and 11-OH-THC) as dependent variables were conducted in the three cannabis-exposed treatment groups. Pearson correlation coefficients were computed to examine the associations between THC concentrations detected in plasma and THC metabolites in each of the three cannabis-exposed treatment groups. Correlations were also computed between number of vapor deliveries earned on day 21 (the day plasma was collected), THC, 11-OH-THC, and THC-COOH in each of the three cannabis-exposed groups. A series of moderation analyses with sex as a moderator indicated no significant sex differences in the correlations between vapor deliveries earned on day 21 and THC and its metabolites. Therefore, these correlations were computed using the two sexes combined.

#### 2.3.3 Basal CORT

A 2 × 2 × 4 mixed factorial ANOVA was used to examine differences in basal CORT concentration (the dependent variable) using sex and treatment condition as between-subjects factors and time (pre-vs. post-vapor self-administration) as a within-subjects factor.

#### 2.3.4 Stress reactivity

Change in CORT concentration was calculated for the pre-vapor stress challenge (post-stress minus pre-stress concentration) as a baseline measure of stress reactivity, with higher scores indicating greater stress reactivity. Change in stress reactivity, from before to after vapor self-administration, was then computed by subtracting pre-vapor stress reactivity from post-vapor stress reactivity. As such, negative scores indicate greater reactivity prior to vapor self-administration while positive scores indicate greater reactivity after vapor self-administration. Changes in stress reactivity were analyzed using a 2 × 2 × 4 between-groups ANOVA, with change in stress reactivity from pre-to post-vapor self-administration as the dependent variable and stress, sex, and treatment as independent variables. Given that we only expected to find blunted stress reactivity in the stress exposed animals, we further performed separate 2 × 4 ANOVAs in the animals exposed to the post-vapor stress challenge and the no stress control animals with sex and treatment as independent variables and change in stress reactivity as the dependent variable. Interactions with sex were probed by conducting one-way ANOVAs examining effects of treatment on change in stress reactivity in males and females separately. Bonferroni post hoc tests were used to probe significant main effects of treatment.

#### 2.3.5 Baseline stress challenge

Pearson correlations were computed between pre-vapor stress reactivity, vapor deliveries, and active-port responses for each treatment group using averages of the first 10 days of vapor self-administration to examine whether animals that were more reactive to stress at baseline self-administered more cannabis vapor. Cronbach's α was calculated as a measure of reliability/internal consistency in active responding and vapor deliveries over the final 10 days of self-administration. These values were 0.91 and 0.93 respectively, which indicates strong consistency in responses across these days.

## 3 Results

### 3.1 Vapor self-administration

**Fig. 2:**
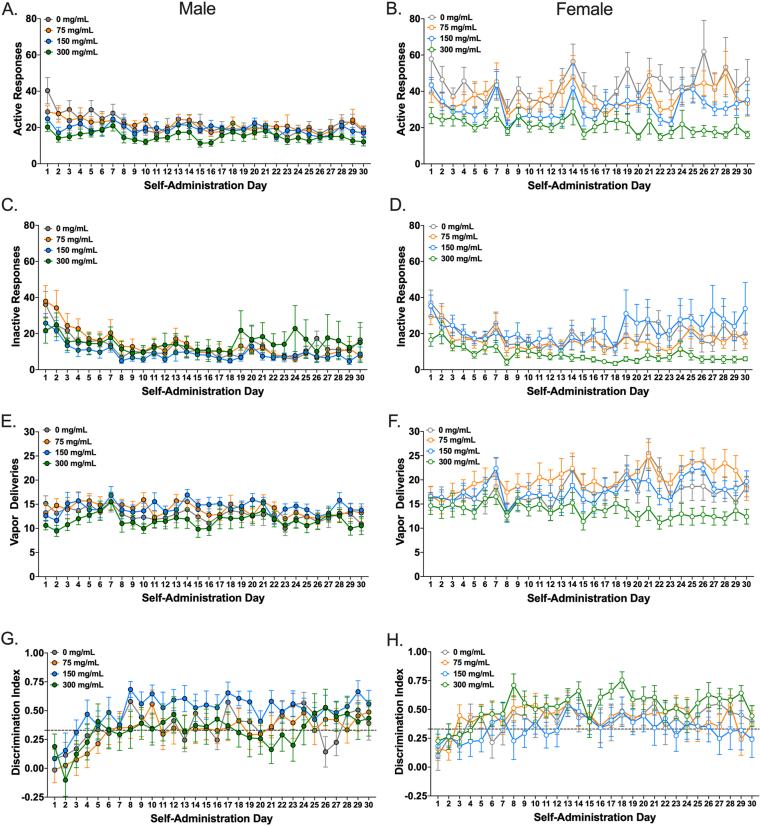
**Adult female rats self-administer cannabis vapor at a greater rate than adult male rats.** Mean (+/− SEM) number of active (**A** and **B**) and inactive (**C** and **D**) nosepoke responses made, vapor deliveries earned (**E** and **F**), and discrimination indices (**G** and **H**) for vapor containing 0 mg/ml (grey), 75 mg/ml (orange), 150 mg/ml (blue), or 300 mg/ml (green) cannabis extract. Rats were trained to respond on an FR-1 schedule of reinforcement for all 30 days of self-administration. Active and inactive responding panels include responses made during the 60 s timeout period, which had no programmed consequences. Male rats (left) are denoted with closed circles, while female rats (right) are denoted with open circles (n = 13/sex/group). (For interpretation of the references to colour in this figure legend, the reader is referred to the Web version of this article.)

The ANOVA for active-port responses indicated a significant three-way interaction, *F*(30.82, 986.41) = 1.55, *p* = .029, $\eta_{\text{p}}^{2}$ = 0.05. Therefore, follow-up ANOVAs were conducted separately for each sex. These analyses indicated a significant effect of time in male rats, meaning that responding differed across self-administration days, *F*(8.99, 431.94) = 4.80, *p* < .001 $\eta_{\text{p}}^{2}$ = 0.09 but the effect of treatment and the time × treatment interaction were not significant. As shown in Fig. 2A, active-port responses in male rats generally decreased over time. There was also a significant effect of time in female rats, *F*(8.58, 412.10) = 4.31, *p* < .001, $\eta_{\text{p}}^{2}$ = 0.08, as well as a significant effect of treatment, *F*(3, 48) = 4.50, *p* = .007, $\eta_{\text{p}}^{2}$ = 0.22, but the time × treatment interaction was not significant (Fig. 2B). Bonferroni post hoc tests on the effect of treatment indicated that female rats in the 300 mg/ml condition (*M* = 21.07, *SD* = 7.78) had significantly fewer responses than those in the 0 mg/ml conditions (*M* = 44.17, *SD* = 22.05, *p* = .005, *d* = 1.40). No other contrasts were statistically significant.

In contrast, the ANOVA for inactive-port responses showed no significant interactions or main effects of sex or treatment. There was only a significant effect of time, *F*(6.54, 627.52) = 11.76, *p* < .001, $\eta_{\text{p}}^{2}$ = 0.11, with inactive-port responses generally decreasing over time for both sexes (Fig. 2C and D).

The ANOVA for vapor deliveries also indicated a significant three-way interaction, *F*(34.47, 1103.06) = 1.56, *p* = .021, $\eta_{\text{p}}^{2}$ = 0.05. After splitting the data by sex, a significant effect of time on the number of vapor deliveries earned in male rats was found, *F*(8.05, 386.42) = 2.77, *p* = .005, $\eta_{\text{p}}^{2}$ = 0.06, but the effect of treatment and the time × treatment interaction were not significant (Fig. 2E). To follow-up on the main effect of time, the mean number of vapor deliveries in the first 10 days vs. last 10 days were compared. However, no significant difference was detect in the males, *t*(51) = 1.54, *p* = .129, *d* = 0.21. In contrast, there was a significant time × treatment interaction in female rats, *F*(34.36, 549.70) = 1.56, *p* = .024, $\eta_{\text{p}}^{2}$ = 0.09. Subsequent comparisons indicated a significant increase in responses over time for females in the 0 mg/ml (*F*(29, 348) = 2.39, *p* < .001, $\eta_{\text{p}}^{2}$ = 0.17), 75 mg/ml (*F*(29, 348) = 2.63, *p* < .001, $\eta_{\text{p}}^{2}$ = 0.18), and 150 mg/ml (*F*(29, 348) = 2.60, *p* < .001, $\eta_{\text{p}}^{2}$ = 0.18) conditions, but no change over time for females in the 300 mg/ml condition (*F*(29, 348) = 1.12, *p* = .304, $\eta_{\text{p}}^{2}$ = 0.09) (Fig. 2F).

Finally, the ANOVA for the discrimination index showed no significant interactions or main effects of sex or treatment. There was only a significant main effect of time, *F*(15.45, 1483.23) = 10.12, *p* < .001, $\eta_{\text{p}}^{2}$ = 0.10, with the discrimination index increasing over time (Fig. 2G and H).

### 3.2 Plasma THC and metabolite concentrations

**Fig. 3:**
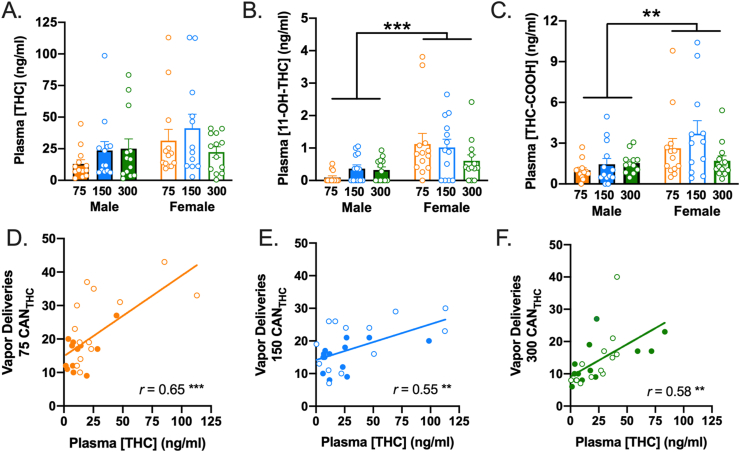
**Cannabis vapor self-administration produces biologically relevant concentrations of THC and metabolites in plasma.** (A) The mean concentration of THC present in plasma did not differ significantly across sexes or treatment groups. (**B** and **C**) The mean concentration of **(B)** 11-OH-THC and **(C)** THC-COOH was significantly greater in female rats relative to male rats, irrespective of treatment condition. Error bars represent SEM. The concentration of THC was significantly and positively correlated with the number of vapor deliveries earned for rats in the **(D)** 75 mg/ml, **(E)** 150 mg/ml, and **(F)** 300 mg/ml conditions. Male rats are denoted with closed circles, while female rats are denoted with open circles on the scatterplots. n = 13/sex/group ** denotes *p* < .01, *** denotes *p* < 001.

The ANOVA examining effects of treatment and sex on THC in the three cannabis-exposed groups indicated no significant main effects of treatment or sex and no treatment × sex interaction (Fig. 3A). There were also no significant main effects of treatment or sex × treatment interactions on the two metabolites. However, there were main effects of sex on THC-COOH, *F*(1, 72) = 8.87, *p* = .004, $\eta_{\text{p}}^{2}$ = 0.17, and 11-OH-THC, *F*(1, 72) = 18.38, *p* < .001, = 0.20. As depicted in Fig. 3B and **C,** females had significantly higher levels of the two metabolites than males. As shown in Table 1, there were large, positive, statistically significant correlations between THC and its metabolites in the 75 mg/ml and 150 mg/ml groups. In contrast, the 300 mg/ml group only demonstrated significant correlations between the two metabolites (THC-COOH and 11-OH-THC). Moreover, there were large, positive, statistically significant correlations between the number of vapor deliveries earned on day 21 and THC as well as its metabolites in all three experimental groups, with the exception of the correlation between 11-OH-THC and vapor deliveries in the 300 mg/ml group, which was null (see Table 1 & Fig. 3D–F).

**Table 1:** **Correlations between THC, THC-COOH, 11-OH-THC, and vapor deliveries**.

	**THC**	**THC-COOH**	**11 OH-THC**
**75 mg/ml (N = 26)**			
THC-COOH	.50**		
11-OH-THC	.59**	.93***	
Day 21 # Vapor Deliveries	.65***	.51**	.61***
**150 mg/ml (N = 26)**			
THC-COOH	.69***		
11-OH-THC	.65***	.92***	
Day 21 # Vapor Deliveries	.55**	.67***	69***
**300 mg/ml (N = 26)**			
THC-COOH	.28		
11-OH-THC	.24	.85***	
Day 21 # Vapor Deliveries	.58**	.43*	.35

*p < .05, **p < .01, ***p < .001.

### 3.3 Basal corticosterone

**Fig. 4:**
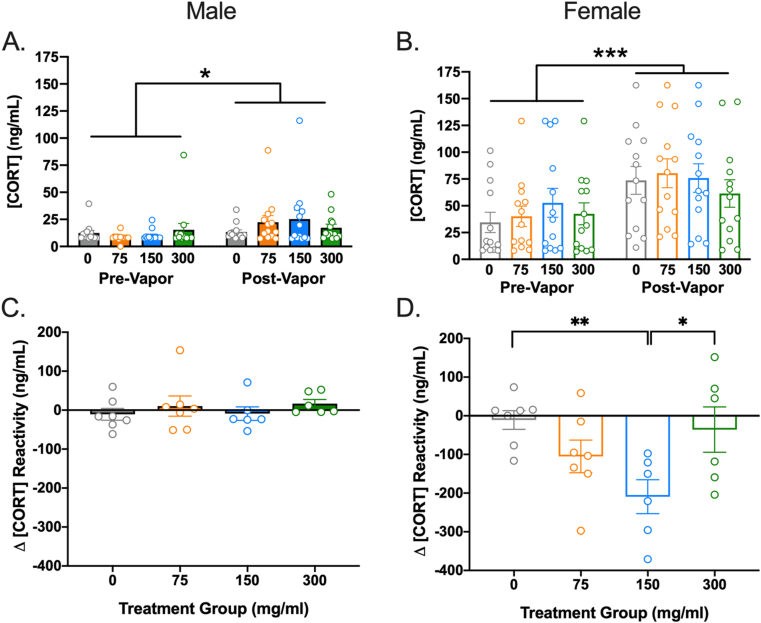
**Cannabis vapor self-administration dose-dependently dampens stress reactivity in female rats.** The mean plasma concentration of CORT was significantly higher at baseline after 30 days of vapor self-administration relative to the pre-vapor timepoint for both **(A)** male and **(B)** female rats, irrespective of treatment condition. n = 13/sex/group, *p < .05, *** denotes *p* < .001. **(C)** The mean change in stress-induced CORT reactivity from pre-vapor to post-vapor was not significantly different in male rats for any treatment condition. **(D)** Conversely, the mean change in stress-induced CORT reactivity was significantly reduced in female rats that self-administered the 150 mg/ml cannabis extract compared to female rats that self-administered vehicle (0 mg/ml) or 300 mg/ml cannabis vapor. Negative scores indicate greater reactivity prior to vapor self-administration while positive scores indicate greater reactivity after vapor self-administration. n = 6–7/sex/group, *p < .05, ** denotes *p* < .01 significant differences in stress reactivity change scores. Error bars represent SEM.

The ANOVA on basal CORT indicated no significant effect of treatment, *F*(3, 96) = 0.55, *p* = .647,$\eta_{\text{p}}^{2}$ = 0.02. However, there were significant effects of sex, *F*(1, 96) = 80.64, *p* < .001, $\eta_{\text{p}}^{2}$ = 0.46 – with females showing higher basal CORT – and time, *F*(1, 96) = 19.15, *p* < .001, $\eta_{\text{p}}^{2}$ = 0.17 – with basal CORT increasing from before to after vapor exposure. The interaction between time and sex was also significant, *F*(1, 96) = 6.42, *p* = .013, $\eta_{\text{p}}^{2}$ = 0.06. No other interactions were significant. The sex × time interaction was probed by examining the main effects of time (pre vs. post-vapor exposure) in males and females separately. A paired samples *t*-test indicated a significant increase in basal CORT from before (*M* = 42.49, *SD* = 38.36) to after (*M* = 72.87, *SD* = 46.57) vapor self-administration in females, *t*(51) = -3.79, *p* < .001, *d* = 0.70 (Fig. 4A). Males also demonstrated a significant increase in basal CORT from before (*M* = 11.52, *SD* = 11.72) to after (*M* = 19.61, *SD* = 19.76) vapor self-administration, *t*(51) = -2.43, *p* = .019, *d* = 0.50 (Fig. 4B). However, the significant sex × time interaction indicates that the magnitude of the increase was significantly smaller in males than in females.

### 3.4 Stress reactivity

The ANOVA using change in stress reactivity as the dependent variable indicated significant main effects of sex, *F*(1,88) = 23.23, *p* < .001, $\eta_{\text{p}}^{2}$ = 0.21, stress, *F*(1,88) = 5.74, *p* = .02,$\eta_{\text{p}}^{2}$ = 0.06, and treatment, *F*(3, 88) = 3.04, *p* = .017, $\eta_{\text{p}}^{2}$ = 0.09. The sex × treatment interaction, *F*(3,88) = 2.49, *p* = .066, $\eta_{\text{p}}^{2}$ = 0.08, and sex x stress × treatment interaction, *F*(3,88) = 2.17, *p* = .098, $\eta_{\text{p}}^{2}$ = 0.07 were not statistically significant but indicated trends. For the no stress control group, follow-up ANOVAs indicated a significant effect of sex, *F*(1,44) = 8.48, *p* = .006, $\eta_{\text{p}}^{2}$ = 0.16, with females demonstrating greater overall change in CORT than males. However, the effect of treatment, *F*(3,44) = 1.16, *p* = .337, $\eta_{\text{p}}^{2}$ = 0.07 and sex × treatment interaction were not significant in the no-stress control group, *F*(3,44) = 1.38, *p* = .261, $\eta_{\text{p}}^{2}$ = 0.09, In contrast, for the group exposed to the post-vapor stress challenge there were significant main effects of sex, *F*(1, 44) = 15.50, *p* < .001, $\eta_{\text{p}}^{2}$ = 0.26, and treatment, *F*(3, 44) = 3.80, *p* = .017, $\eta_{\text{p}}^{2}$ = 0.21, as well as a sex × treatment interaction, *F*(3, 44) = 3.36, *p* = .027, $\eta_{\text{p}}^{2}$ = 0.19. Follow-up ANOVAs in males and females separately suggested no significant treatment effect in males, *F*(3,22) = 0.52, *p* = .672, $\eta_{\text{p}}^{2}$ = 0.07 (Fig. 4C). In females, however, there was a significant effect of treatment, *F*(3,22) = 4.17, *p* = .018, $\eta_{\text{p}}^{2}$ = 0.36. Bonferroni post-hoc tests indicated that females in the 150 mg/ml condition had lower stress reactivity post-vapor self-administration (*M* = −209.27, *SD* = 107.32) than the 0 mg/ml (*M* = −11.00, *SD* = 64.07, *p* = .021, *d* = 2.24) condition (Fig. 4D). There were also non-significant trends wherein females in the 150 mg/ml condition exhibited lower post-vapor stress reactivity than females in the 300 mg/ml condition (*M* = −35.79, *SD* = 143.72, *p* = .068, *d* = 1.37). Thus, there was blunted stress reactivity following vapor self-administration among female rats that self-administered the 150 mg/ml cannabis vapor.

### 3.5 Baseline stress challenge

As shown in Table 2, correlations between pre-vapor stress reactivity, vapor deliveries, and active-port responses for each treatment condition over the last 10 days of vapor self-administration indicated no significant relationships between pre-vapor stress reactivity and active-port responses or vapor deliveries in any of the cannabis groups. These results contradict the alternative hypothesis that lower stress reactivity represents a risk factor for cannabis use.

**Table 2:** **Correlations between pre-vapor stress reactivity and mean vapor deliveries and active responses in the first 10 days of self-administration**.

	Vapor Deliveries		Active Responses
	*r*	*r*	
300 mg/mL	.068	.036	
150 mg/mL	-.045	.083	
75 mg/mL	.095	.135	
0 mg/mL	.048	.341	

## 4 Discussion

The primary purpose of this study was to examine alterations in stress reactivity and basal CORT after 30 days of cannabis vapor self-administration. We hypothesized that cannabis vapor self-administration would produce sex and dose-dependent alterations in stress reactivity and basal CORT. Ultimately, results from this study indicate that repeated cannabis exposure dose-dependently dampens stress reactivity in female (but not male) rats. Further, blunted stress reactivity at baseline did not predict vapor self-administration, which contradicts the alternative hypothesis that lower stress reactivity represents a risk factor for cannabis use. Additionally, basal CORT was found to increase following vapor self-administration in both sexes. However, this increase in basal CORT occurred in all treatment conditions, including vehicle, and was thus not attributed to effects of cannabis exposure *per se*.

### 4.1 Cannabis self-administration dampens stress reactivity in females

Our results specifically indicated a blunted stress response among female rats that self-administered 150 mg/ml cannabis vapor compared to females that self-administered 0 mg/ml vehicle vapor, as well as a similar non-significant trend for rats that self-administered 300 mg/ml cannabis vapor. In contrast, blunted stress reactivity at baseline did not predict cannabis vapor self-administration. These findings support our original hypothesis and are consistent with findings of blunted stress reactivity in human cannabis users in response to a multidimensional stressor (Cuttler et al., 2017) and unpleasant images (Somaini et al., 2012), as well as decreased amygdala activation in response to images of threatening faces (Cornelius et al., 2010). However, since it is unethical to manipulate chronic cannabis use in humans, establishing the directionality of this effect has been difficult. This is the first study to demonstrate such an effect in rodents, which is important because rodents were randomly assigned to receive cannabis or control vapor. The high degree of control afforded by the rodent self-administration model therefore provides the first evidence of an altered stress response *caused* by cannabis vapor self-administration.

Although there is converging evidence of an altered neuroendocrine response in sober, chronic cannabis users, it is unclear what mechanisms are responsible for these changes. CB1 receptors are densely expressed in regions of the brain that regulate the emotional and neuroendocrine response to stressors (e.g., amygdala, hippocampus, medial prefrontal cortex; Glass et al., 1997), and the ECB system typically suppresses hypothalamic-pituitary-adrenal (HPA) axis activation until the onset of a stressor (Gray et al., 2015). Therefore, it could be that residual THC, as a result of chronic cannabis use, interferes with the reduction in ECB signaling associated with disinhibition of the HPA axis, thereby constraining normal stress-induced HPA axis activation and dampening the appropriate downstream release of CORT. Indeed, we have recently shown that THC remains detectable in the brains of rats trained to self-administer THC-rich cannabis vapor for up to 24 h after their final self-administration session (Freels et al., 2020).

### 4.2 Sex differences in cannabis self-administration and HPA axis activity

This study is the first to use both male and female rats to explore sex differences in cannabis vapor self-administration and stress reactivity. HPA axis responsivity differs considerably across sexes, and gonadal hormones are known to play an important role in the regulation of the HPA axis (see Oyola and Handa 2017 for review). Female rats exhibit a more robust ACTH response to stress (Young 1996), as well as greater CORT increases following a stressor compared to males (Figueiredo et al., 2002). Women are also more likely to experience stress-related mental illnesses than men (American Psychiatric Association, 2013) and women are more likely to use cannabis to cope with symptoms of anxiety (Cuttler et al., 2016). Despite these concerns, females are often underrepresented in animal research and as a result, much less is known about the effects of cannabis use in females. Our data indicate significant sex differences in basal CORT, responding for cannabis vapor, and the effects of cannabis vapor on stress reactivity and changes in basal CORT. Overall, female rats self-administered more vapor than male rats, which likely contributes to the sex differences in alterations in stress reactivity observed and is consistent with other studies demonstrating higher levels of intravenous self-administration of the CB1 receptor agonist WIN55,212-2 in female rats compared to males (Fattore et al., 2009, Fattore et al., 2010).This is also consistent with recent data collected in our laboratory indicating that adolescent female rats self-administer cannabis vapor at a significantly higher rate than adolescent male rats (Freels et al., 2020). However, the opposite trend is true in human users, where men typically use cannabis more frequently and in higher quantities than women (Cuttler et al., 2016).

These sex differences in cannabis self-administration complicate interpretation of our stress reactivity data, as the blunted stress reactivity observed only in females may be attributed to the quantity of THC they were exposed to, rather than sex *per se*. Consistent with this alternative interpretation, female rats demonstrated significantly higher levels of the two primary metabolites of THC (THC-COOH and 11-OH-THC). There is also evidence that female rats exhibit higher metabolite levels even when receiving the same intraperitoneal dose of THC as males (Narimatsu et al., 1991; Tseng et al., 2004). Thus, a parsimonious interpretation of these data is that female rats respond for more vapor deliveries because they may be metabolizing the drug at a faster rate. In future studies it will be important to address whether the observed effects on stress reactivity were perhaps due to sex differences in the metabolism of THC, or rather due to differences in rates of cannabis vapor self-administration. Future studies where male rats are yoked to females could directly address whether effects are due to differences in the amount of cannabis exposure or bona fide sex differences in the effects of cannabis on the stress response.

### 4.3 Dose-dependent effects of a high-THC cannabis extract

This study is also the first to demonstrate dose-dependent responding for a highly potent cannabis concentrate preparation in rats. Specifically, our data show that female (but not male) rats receiving the most concentrated preparation (300 mg/ml) made, on average, fewer active responses per day (*M* = 21.07) relative to those receiving the 150 mg/ml (*M* = 31.14) or 75 mg/ml (*M* = 36.37) cannabis preparations. These data suggest that rats show a greater preference for less concentrated cannabis extract and thus scale their responding to self-titrate their THC exposure that effectively constrains circulating THC levels within a desired range. This is consistent with emerging human data demonstrating that cannabis users inhaling high potency cannabis concentrates take on average significantly fewer puffs (M_[THC]_ = 73%; M_puffs_ = 6.5) than cannabis users inhaling lower potency cannabis flower (M_[THC]_ = 23%; M_puffs_ = 17.5) (Cuttler and LaFrance, 2019).

Although THC and metabolite levels were similar across treatment groups, this is likely because we measured plasma THC on a single day (day 21) and at an arbitrary timepoint (at the end of the 60 min session), rather than at peak intoxication, which likely occurred earlier in the session. Since THC and metabolite concentrations are dynamic following this route of administration and largely dependent on the pattern of self-administration, it is likely that our measurements missed peak plasma THC levels, which we suspect would be a better read-out of total THC exposure. Accordingly, we have documented a loading dose phenomenon for cannabis vapor self-administration such that rats exhibit the bulk of responding during the initial 15 min bin of the session (Freels et al., 2020). Pharmacokinetic studies employing measures of plasma THC at several time points during self-administration would provide better insight into the time course of plasma THC and allow us to determine whether *peak THC concentrations* are also similar for these dose groups. We suspect that this would be unlikely however, since plasma THC and the longer-lasting THC-COOH metabolite were highest in the female group that self-administered the 150 mg/ml cannabis preparation.

Notably, blunted stress reactivity was only observed in female rats that self-administered the medium concentration cannabis preparation (150 mg/ml), which also produced the highest mean levels of THC following self-administration on day 21. This may be attributed to the greater variability in responding observed in female rats. Specifically, we found that female rats self-administering the 150 mg/ml and 75 mg/ml cannabis extracts showed increases in vapor self-administration over time. The lack of blunted stress reactivity observed in females receiving the high dose preparation was thus likely due to the lack of escalation in dose and the lower number of vapor deliveries earned in this group. Conversely, despite having the highest rates of responding among cannabis-exposed groups, rats receiving 75 mg/ml cannabis vapor may not have exhibited blunted stress reactivity because of the relatively low concentration of THC present in this extract that ultimately did not elicit THC concentrations sufficient to cause these stress-related adaptations. However, it should be noted that any interpretation of dose-specific effects is complicated by sources of variability that are inherent to a response-contingent vapor delivery approach, such as individual differences in rates of responding and the relative position of the animal in the chamber when each puff is delivered. It remains unknown whether volitional exposure to cannabis is necessary for these effects, or whether passive cannabis vapor delivery is similarly capable of eliciting blunted stress reactivity.

### 4.4 Vapor self-administration non-specifically increases basal CORT concentration

Although both male and female rats showed a significant increase in basal CORT from before to after vapor self-administration, basal CORT was not significantly different across the treatment groups, which was contrary to our prediction that cannabis exposure would selectively increase basal CORT. This finding is consistent with some research on humans failing to demonstrate increased basal CORT in cannabis users (Block et al., 1991; Cloak et al., 2015; Cuttler et al., 2017; Lisano et al., 2019), but contradicts a number of other studies demonstrating heightened basal (Carol et al., 2017; King et al., 2011; Somaini et al., 2012) and awakening cortisol (Huizink et al., 2006; Monteleone et al., 2014) in human cannabis users relative to non-users. However, it is noteworthy that two of the latter studies are confounded by inclusion of participants with schizophrenia or at risk for psychosis (Carol et al., 2017; Monteleone et al., 2014). The finding that basal CORT increased across all treatment conditions indicates that either vapor exposure produces effects on basal CORT that are similar to the effects of cannabis, or more likely, that basal CORT simply increased across time, perhaps due to age, stress, or experimenter handling. Since blood sampling occurred 24 h after the last self-administration session, this increase may also be an artifact of anticipatory arousal as a result of circadian alignment with daily vapor self-administration each morning. Future research will need to examine these possibilities by employing control groups that are not exposed to vapor or experimenter handling for the duration of self-administration training.

### 4.5 Implications and conclusions

The implications of blunted stress reactivity remain a matter of debate. On one hand, cannabis-induced reductions in CORT reactivity could seemingly protect against the detrimental effects of chronic stress by preventing excessive glucocorticoid activity that can lead to atrophy in brain regions responsible for the physiological and emotional response to stress (see McEwen et al., 2016 for review). As such, this blunted stress response could impart resilience to stress-related disorders that are characterized by an overreactive HPA axis and persistent hyperarousal. On the other hand, research indicates that using cannabis to cope with stress is associated with several negative outcomes, including cannabis use problems (Lee et al., 2007; Simons et al., 2005), negative affect (Gobbi et al., 2019; Twomey 2017), poorer mental health, greater risk for pathology, and increased levels of distress (Brodbeck et al., 2007). Accordingly, previous research indicates that an onset of symptoms of schizophrenia preceded by cannabis use was accompanied by cortisol dysregulation, while patients with a diagnosis of schizophrenia that did not use cannabis had normal cortisol responses (Gorka et al., 2016). Further, the atypical subtype of major depression has been associated with reduced cortisol reactivity compared to healthy controls (Gold and Chrousos 2002). Despite medical cannabis users reporting acute antidepressant effects from cannabis, repeated cannabis use may actually exacerbate depression over time (Cuttler et al., 2018; Gobbi et al., 2019; Lev-Ran et al., 2014). Additional research into the effects of cannabis vapor self-administration will be necessary for determining the biological mechanisms underlying cannabis-induced perturbations of the stress response, as well as the long-term consequences of blunted stress reactivity on various aspects of mental health.

## CRediT authorship contribution statement

**Nicholas C. Glodosky:** Formal analysis, Writing - original draft, Visualization. **Carrie Cuttler:** Conceptualization, Formal analysis, Supervision, Writing - review & editing. **Timothy G. Freels:** Investigation. **Hayden R. Wright:** Investigation. **Manuel J. Rojas:** Investigation. **Samantha L. Baglot:** Investigation. **Matthew N. Hill:** Methodology, Resources. **Ryan J. McLaughlin:** Conceptualization, Methodology, Funding acquisition, Supervision, Writing - review & editing, Project administration.

## Declaration of competing interest

The authors state no competing financial interests or other interests that might be perceived to influence the results and discussion reported in this paper.
